# Insights into the origin of DNA methylation differences between monozygotic twins discordant for schizophrenia

**DOI:** 10.1186/s40303-015-0013-5

**Published:** 2015-06-26

**Authors:** Melkaye G. Melka, Christina A. Castellani, Richard O’Reilly, Shiva M. Singh

**Affiliations:** Molecular Genetics Unit, Western Science Centre, Department of Biology, The University of Western Ontario, London, Ontario N6A 5B7 Canada; Department of Psychiatry, The University of Western Ontario, London, Ontario N6A 5B7 Canada

## Abstract

**Background:**

DNA methylation differences between monozygotic twins discordant for schizophrenia have been previously reported. However, the origin of methylation differences between monozygotic twins discordant for schizophrenia is not clear. The findings here argue that all DNA methylation differences may not necessarily represent the cause of the disease; rather some may result from the effect of antipsychotics.

**Methods:**

Methylation differences in rat brain regions and also in two pairs of unrelated monozygotic twins discordant for schizophrenia have been studied using genome-wide DNA methylation arrays at Arraystar Inc. (Rockville, Maryland, USA). The identified gene promoters showing significant alterations to DNA methylation were then further characterized using ingenuity pathway analysis (Ingenuity System Inc, CA, USA).

**Results:**

Pathway analysis of the most significant gene promoter hyper/hypomethylation revealed a significant enrichment of DNA methylation changes in biological networks and pathways directly relevant to neural development and psychiatric disorders. These included HIPPO signaling (*p* = 3.93E-03) and MAPK signaling (*p* = 4.27E-03) pathways involving hypermethylated genes in schizophrenia-affected patients as compared to their unaffected co-twins. Also, a number of significant pathways and networks involving genes with hypomethylated gene promoters have been identified. These included *CREB* signaling in neurons (*p* = 1.53E-02), Dopamine-*DARPP32* feedback in cAMP signaling (*p* = 7.43E-03) and Ephrin receptors (*p* = 1.13E-02). Further, there was significant enrichment for pathways involved in nervous system development and function (*p* = 1.71E-03-4.28E-02).

**Conclusion:**

The findings highlight the significance of antipsychotic drugs on DNA methylation in schizophrenia patients. The unique pathways affected by DNA methylation in the two pairs of monozygotic twins suggest that patient-specific pathways are responsible for the disease; suggesting that patient-specific treatment strategies may be necessary in treating the disorder. The study reflects the need for developing personalized medicine approaches that take into consideration epigenetic variations between patients.

**Electronic supplementary material:**

The online version of this article (doi:10.1186/s40303-015-0013-5) contains supplementary material, which is available to authorized users.

## Background

Epigenetic modifications may affect gene expression without the existence of changes in DNA sequence [1]. They are known to affect a number of neurological disorders [2, 3] including psychosis [4]. Specifically, DNA methylation is known to affect gene expression via transcriptional regulation [5]. Further, a number of environmental factors may affect gene-specific DNA methylation, and this alteration may be maintained during genetic transmission through mitosis and meiosis [6]. These dynamic changes may explain the discordance of monozygotic twins [7] that are considered genetically identical. In fact, several studies have reported methylation differences between monozygotic twins discordant for a variety of disorders including schizophrenia [8, 9]. Interestingly, hypermethylation has been reported to affect genes that have been implicated in psychiatric disorders [10]. These results have been used to argue that aberrations in gene specific DNA methylation must represent, at least, one of the contributing mechanisms in the manifestation of psychiatric disorders [10]. The epigenetic float and drift during development can be stochastic, caused by environmental factors or (most likely) the result of a combination of the two [11]. Although the involvement of stochastic processes have been difficult to establish, a number of environmental agents are known to affect epigenetic-dependent disease phenotypes in a number of disorders. An example of this includes the hypermethylation of tumor suppressor genes in a variety of cancers [12]. Such results question the cause of differentially methylated psychosis-related genes in monozygotic twins discordant for psychosis. There is a possibility that the observed methylation difference is caused by (still unknown) environment factors, such as drugs. This suggests that some of the observed differential methylation between monozygotic twins discordant for this disorder may not necessarily be the cause of the disorder in the affected twin.

In this report, we discuss two complementary sets of results. The first deals with identification of hypermethylation/hypomethylation of a set of genes in the affected individuals of two sets of unrelated monozygotic twins discordant for schizophrenia. The results support the literature that monozygotic twins do differ in the DNA methylation status of their genes and the hypermethylated/hypomethylation genes may take part in pathways affecting psychiatric disorders. Our second set of results shows that an antipsychotic drug (Olanzapine) treatment may cause its effect via hypermethylation/hypomethylation of a number of genes that have been implicated in psychosis [13]. Interestingly, several genes affected by hyper/hypomethylation in the second study are psychosis related genes. Taken together, the results offer novel insight into the epigenetic cause(s) and treatment of psychotic disorders. It forms the focus of this report.

## Methods

This study involves two psychosis-affected monozygotic twins and their healthy co-twins (*n* = 4). All subjects gave written informed consent to participate in the study and contributed whole blood samples for DNA analysis. The demographic information and the medication status of the affected twins are described elsewhere [14]. Genomic DNA was extracted from the blood samples using the PerfectPure DNA Blood Kit, following the manufacture’s protocol (http://www.5prime.com). Samples of genomic DNA were sonicated to random fragments in a size range of about 200–1000 bp. Immunoprecipitation of methylated DNA fragments was performed using Biomag^TM^ magnetic beads coupled mouse monoclonal antibody against 5-methylcytidine. The precipitated DNA was eluted and purified by phenol chloroform extraction and ethanol precipitation. The total input and immunoprecipitated DNA were labeled with Cy3- and Cy5-labeled random 9-mers, respectively, and hybridized to the NimbleGen Human DNA Methylation 3x720K Promoter Plus CpG Island Array, which is a multiplex slide with 3 identical arrays per slide and each array contains 27,728 CpG Islands annotated by UCSC and 22,532 well-characterized RefSeq promoter regions (from about −2,440 to +610 bp of the TSSs) totally covered by ~720,000 probes. Scanning was performed with the Axon GenePix 4000B microarray scanner. This methylation protocol was carried out at ArrayStar Inc (Rockville, MD, USA).

The details of olanzapine treatment of experimental rats are described fully elsewhere [15]. Briefly, adult male Sprague–Dawley rats of 12 weeks of age (250 – 300 g) were purchased from Charles River, PQ, Canada. Rats were weighed and divided into two treatment groups (olanzapine treated and control) with comparable means of weight. Rats received injections of olanzapine (Zyprexa, Lilly, IN, USA; 2.5 mg/kg, i.m.; *n* = 8) or vehicle (phosphate buffered saline (PBS); *n* = 8) between 1:30 and 3:00 pm daily for 19 days.

### Methylation enrichment and peak-finding

Raw data generated from scans were normalized (log2-ratio). It followed Median centering, quantile normalization and linear smoothing using Bioconductor packages Ringo, limma, and MEDME. The results were used in a sliding-window (750 bp) peak-finding algorithm provided by NimbleScan v2.5 (Roche-NimbleGen). A one-sided Kolmogorov-Smirnov (KS) test is applied to determine whether the probes are drawn from a significantly more positive distribution of intensity log2-ratios than those in the rest of the array. Each probe receives a log10 *p*-value score from the windowed KS test around that probe. If several adjacent probes rise significantly above a set threshold, the region is assigned to an enrichment peak (EP). The peak data files are generated from the *p*-value data files. NimbleScan detects peaks by searching for at least two probes above a *p*-value minimum cutoff (−log10) of 2. Peaks within 500 bp of each other are merged. Further details of the quality control and normalization procedures in the twins and rat datasets are presented elsewhere [14, 15]. The genome-wide methylation analysis was applied to two unrelated pairs of monozygotic twins discordant for schizophrenia. For microarray data of replicate samples, a T-test was performed between different groups to calculate the *p* values for each probe. Probes with *p* < 0.05 were identified as differentially methylated probes, which were further analyzed to find Differentially Methylated Regions (DMR). Finally, we have applied a more stringent threshold of significant differences in DNA methylation (*p* < 0.001) to filter the genes affected by DNA methylation changes in their promoter regions. It allowed identification of biologically relevant genes showing hypermethylation in schizophrenia-affected twins as compared to their unaffected co-twin.

### DEP analysis using M’ method

When comparing two groups’ differentially enriched regions, the average of the log2-ratio values for each group (Experiment and Control) was obtained and M’ value (defined by the following equation) was calculated for each probe. Then, the NimbleScan sliding-window peak-finding algorithm was rerun on the data to find the differential enrichment peaks (DEP).

$$ \mathrm{M}' = \mathrm{Average}\left({ \log}_2{\mathrm{MeDIP}}_{\mathrm{E}}/{\mathrm{Input}}_{\mathrm{E}}\right)\ \hbox{-}\ \mathrm{Average}\left({ \log}_2{\mathrm{MeDIP}}_{\mathrm{C}}/{\mathrm{Input}}_{\mathrm{C}}\right) $$

The differential enrichment peaks (DEP) called by the NimbleScan algorithm were filtered according to the following criteria:

i). At least one of the two groups has a median (log_2_ MeDIP/Input) > =0.3 and M’ > 0.
ii). At least half of probes in a peak may have coefficient of variability (CV) < = 0.8 in both two groups.

### Pathway analysis

Genes affected by DMRs were analyzed with Ingenuity Pathway Analysis (IPA) for each patient. Also, the genes identified were matched with genes in the schizophrenia gene database (www.szgene.org). Top pathways and networks related to the two pairs are reported here. Finally, we have assessed whether the genes affected by promoter hyper/hypomethylation were among the psychosis related genes listed in Gemma (www.chibi.ubc.ca/Gemma/home.html). Furthermore, IPA was also used to identify the most significant pathways and networks affected by hyper/hypomethylation in the rat brain regions. Similarities and differences between pathways and networks affected by hyper/hypomethylation in the twins and in rat brain regions (hippocampus and cerebellum) exposed to a therapeutic dose of an antipsychotic drug (olanzapine) were assessed (*n* = 8). Moreover, psychosis related genes showing methylation differences between schizophrenia-affected twins and healthy co-twins were identified.

## Results

### A. Monozygotic twins discordant for schizophrenia – Twin Pair 1

#### Pathways and networks affected by hypermethylation in the patient

**Fig. 1:**
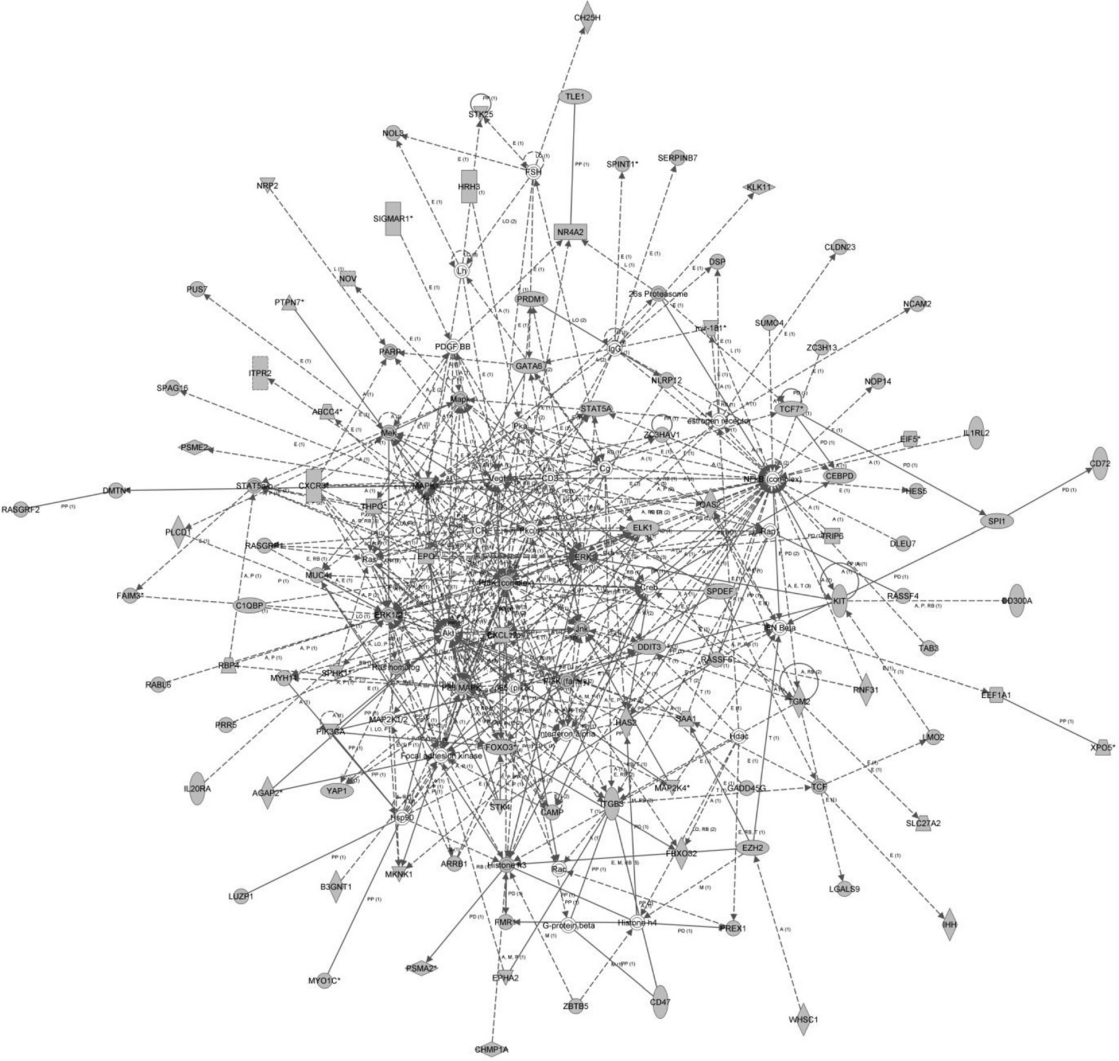
Cellular growth, proliferation, cellular movement & development (genes hypermethylated in the schizophrenia patient in Twin Pair 1 as compared to unaffected co-twin)

Hypermethylated genes in the affected as compared to unaffected twin of twin pair 1 were analyzed with Ingenuity Pathway Analysis (IPA). This analysis identified a number of relevant pathways and networks. These included HIPPO signaling (*p* = 3.93E-03) and MAPK signaling (*p* = 4.27E-03) canonical pathways (Table 1). Also, the diseases and disorders affected by hypomethylation of genes in this patient included developmental and hereditary disorders (*p* = 3.56E-03–4.72E-02). The most significant network affected by hypermethylation of genes in the patient was a cellular growth, proliferation and cellular movement and development network (score = 131) (Fig. 1).

**Table 1:** Most significant pathways and networks identified by IPA of genes showing significant (*p* < 0.001) promoter hypermethylation (A) and hypomethylation (B) of the schizophrenia affected vs. healthy monozygotic twins in twin pair 1

A)		
Canonical Pathways	*P*-value	Ratio^a^
HIPPO signaling	3.93E-03	4/85 (0.047)
MAPK Signaling	4.27E-03	4/87 (0.046)
Molecular and Cellular Functions		
Cell Cycle, DNA Replication, Recombination	1.69E-04–1.51E-02	6
Cellular development, growth and proliferation	1.16E-03–4.45E-02	24
Cell Death and Survival	1.67E-03–4.54E-02	22
Diseases and Disorders		
Developmental disorder	3.56E-03–4.72E-02	16
Hereditary disorder	3.56E-03–2.99E-02	22
Organismal injury and abnormalities	3.56E-03–4.91E-02	45
Most Significant Networks		#of molecules
Cellular Growth, Proliferation, Cellular movement & development		131
Cell Death and Survival, Cellular movement		69
B)		
Canonical Pathways	*P*-value	Ratio^a^
Dopamine-DARPP32 feedback in cAMP signaling	7.43E-03	6/157 (0.038)
Calcium Signalling	1.08E-02	6/170 (0.035)
Ephrin Receptor Signalling	1.13E-02	6/172 (0.035)
Molecular and Cellular Functions		
Gene expression	3.75E-04–4.97E-02	37
Cellular Assembly and Organization	1.15E-03–4.61E-02	22
Small Molecule Biochemistry	3.39E-03–4.51E-02	16
Lipid Metabolism	3.39E-03–3.69E-02	6
Diseases and disorders		# of molecules
Metabolic disease	3.38E-04–4.28E-02	17
Endocrine system disorders	1.95E-04–4.42E-02	21
Nervous system development and function	1.71E-03–4.28E-02	3
Most Significant Networks		
Cell-mediated Immune Response, Cellular Movement		60
Cell Cycle, Cellular Development		56

^a^The number of molecules that reach the threshold of significant methylation difference between twins

(Peak score > =3.1)

#### Pathways and networks affected by hypomethylation in the patient

**Fig. 2:**
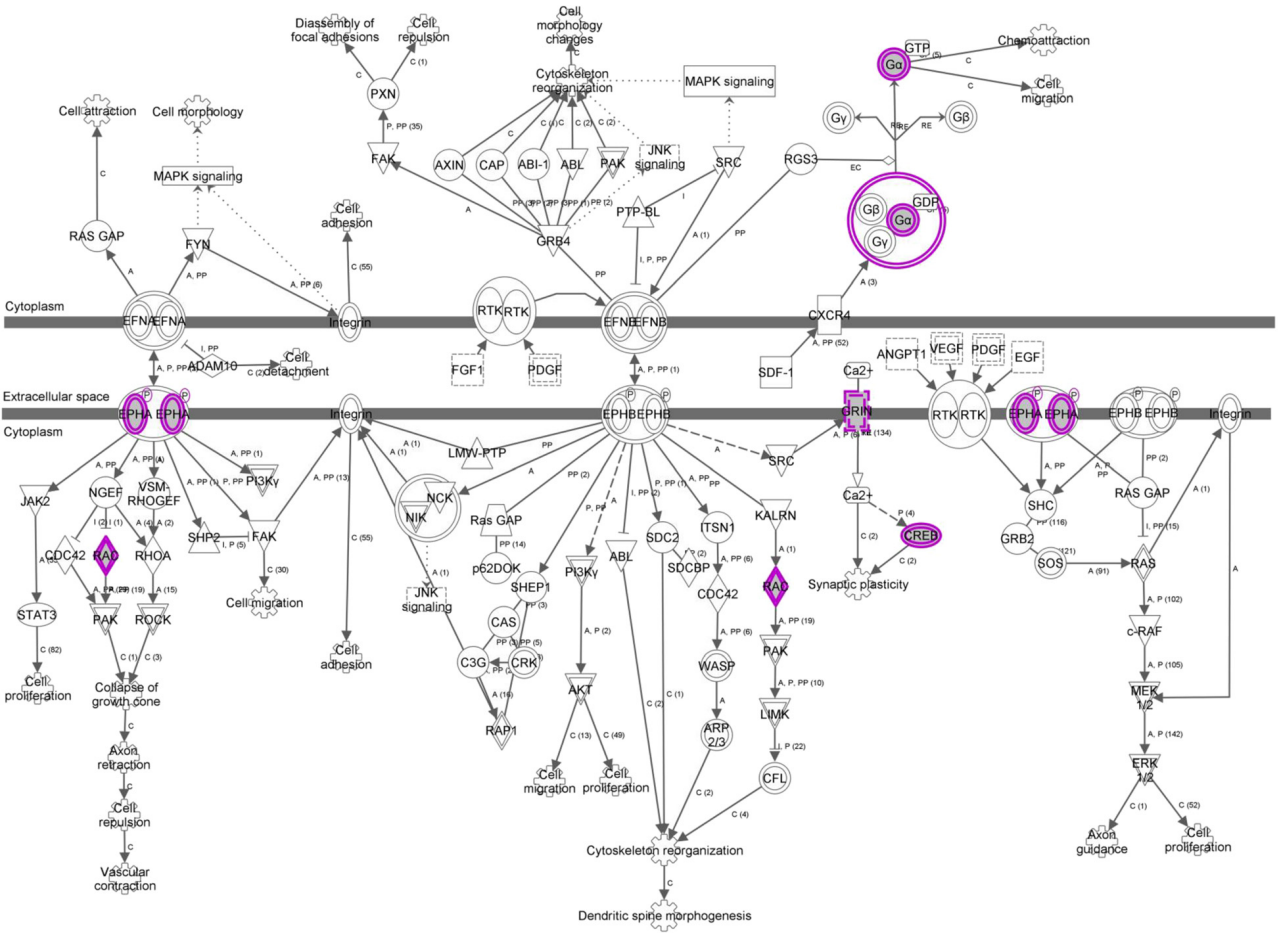
Ephrin receptor signaling pathway (genes hypomethylated in the schizophrenia patient of Twin Pair 1). The genes affected by methylation are highlighted in purple

Among the most significant canonical pathways identified using the list of genes that showed promoter hypomethylation in the patient in twin pair 1 were dopamine-*DARPP32* feedback in cAMP signaling (*p* = 7.43E-03), calcium signaling (*p* = 1.08E-02) and ephrin receptor signaling (*p* = 1.13E-02) (Fig. 2). Also, the most significant molecular and cellular functions revealed using the same list of genes were gene expression (*p* = 3.75E-04–4.97E-02), cellular assembly and organization (*p* = 1.15E-03–4.61E-02), lipid metabolism (*p* = 3.39E-03–3.69E-02), and small molecule biochemistry (*p* = 3.39E-03–4.51E-02). There was also clear enrichment for a pathway involved in nervous system development and function (*p* = 1.71E-03–4.28E-02) (Table 1).

### B. Monozygotic twins discordant for schizophrenia – Twin Pair 2

#### Pathways and networks affected by hypermethylation in the patient

**Fig. 3:**
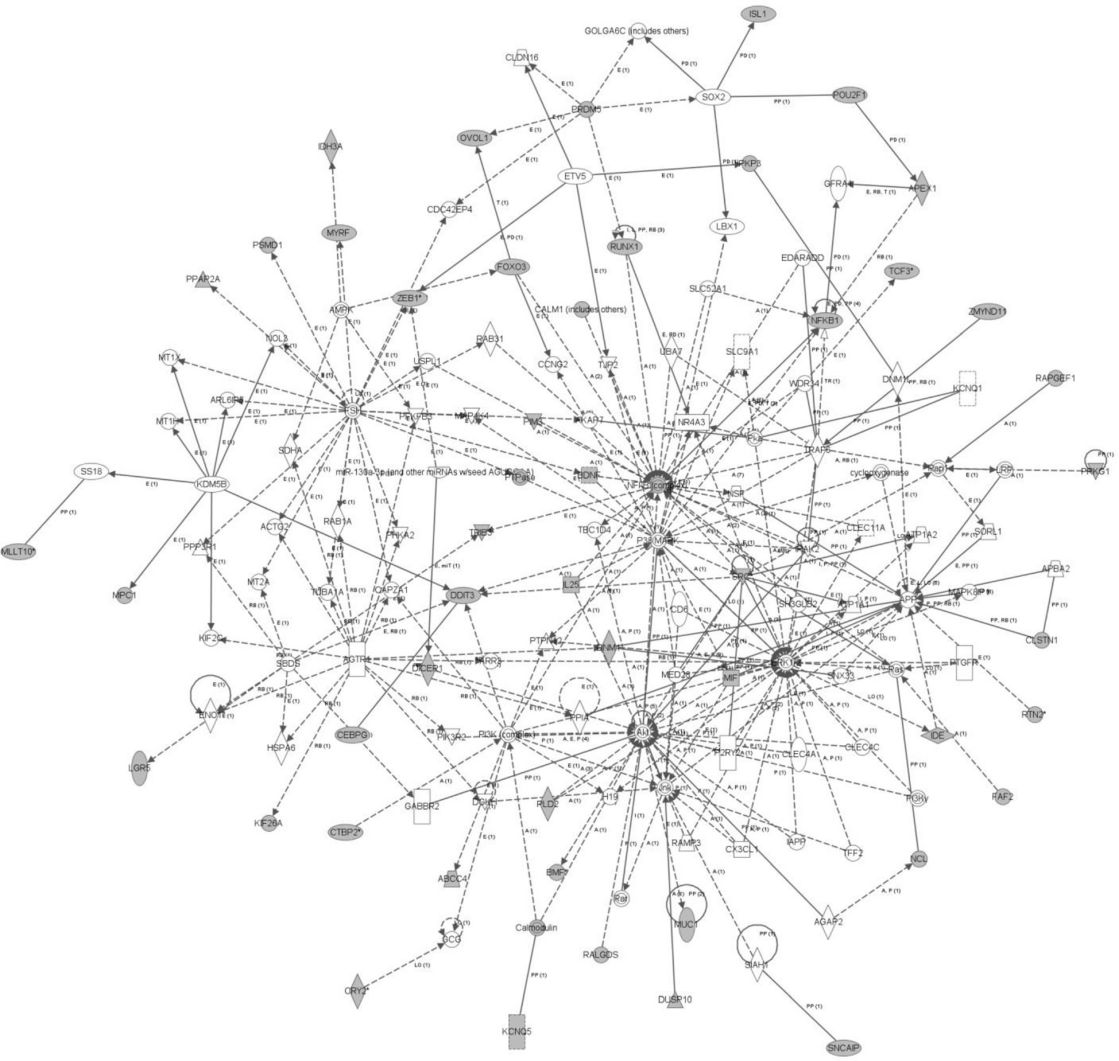
Cell death and survival, cellular growth and proliferation, cell morphology (genes hypermethylated in the schizophrenia patient of Twin Pair 2)

Table 2 presents that the triacylglycerol biosynthesis (*p* = 2.94E-03) pathway was affected by genes that showed promoter hypermethylation in the patient as compared to their unaffected co-twin. These differences also affected gene expression (*p* = 5.79E-06–3.62E-02) and molecular transport and protein trafficking (*p* = 4.01E-03–4.39E-03) functions. The most significant disorders implicated, similarly to twin pair 1, were developmental and hereditary disorders (*p* = 9.84E-04–4.10E-02), organismal injury and abnormalities (*p* = 4.41E-04–4.40E-02), and involved aberrations in gene expression (*p* = 5.79E-06–3.62E-02). The top network affected by hypermethylation of genes included cell death and survival, cellular growth and proliferation, and cell morphology (peak score = 47) (Fig. 3).

**Table 2:** Most significant pathways and networks identified by IPA of genes showing significant (*p* < 0.001) promoter hypermethylation (A) and hypomethylation (B) of the schizophrenia affected vs. healthy monozygotic twins in twin pair 2

A)		
Canonical Pathways	*P*-value	Ratio^a^
Triacylglycerol Biosynthesis	2.94E-03	4/33 (0.121)
Phosphatidylglycerol Biosynthesis II	3.39E-03	3/17 (0.176)
Molecular and Cellular Functions		
Gene Expression	5.79E-06–3.62E-02	53
Cellular Movement	9.84E-04–4.83E-02	19
Molecular transport and protein trafficking	4.01E-03–4.39E-03	16
Diseases and disorders		
Developmental disorder	9.84E-04–4.10E-02	36
Hereditary disorder	9.84E-04–3.77E-02	46
Most Significant Networks		# of molecules
Cell Death and Survival, Cellular Growth		47
Cellular Movement, Immune Cell Trafficking		45
B)		
Canonical Pathways	*P*-value	Ratio^a^
Glutamate Receptor Signaling	1.05E-02	4/56 (0.071)
CREB Signalling in Neurons	1.53E-02	7/169 (0.041)
Molecular and Cellular Functions		
Cell Signalling	4.34E-06–4.51E-02	18
Nucleic Acid Metabolism	4.34E-06–4.51E-02	16
Small Molecular Biochemistry	4.34E-06–4.51E-02	29
Diseases and disorders		
Cell death and survival	3.55E-06–4.76E-02	141
Inflammatory disease	3.35E-03–4.59E-02	18
Most Significant Networks		# of molecules
Cellular Movement, Immune Cell Trafficking		64
Cell Death and Survival, Cell Cycle, Embryonic Development		45
Cellular Movement, Cellular Growth and Proliferation, cell morphology		44

^a^The number of molecules that reach the threshold of significant methylation difference between twins

(Peak score > =3.1)

#### Pathways and networks affected by hypomethylation in the patient

**Fig. 4:**
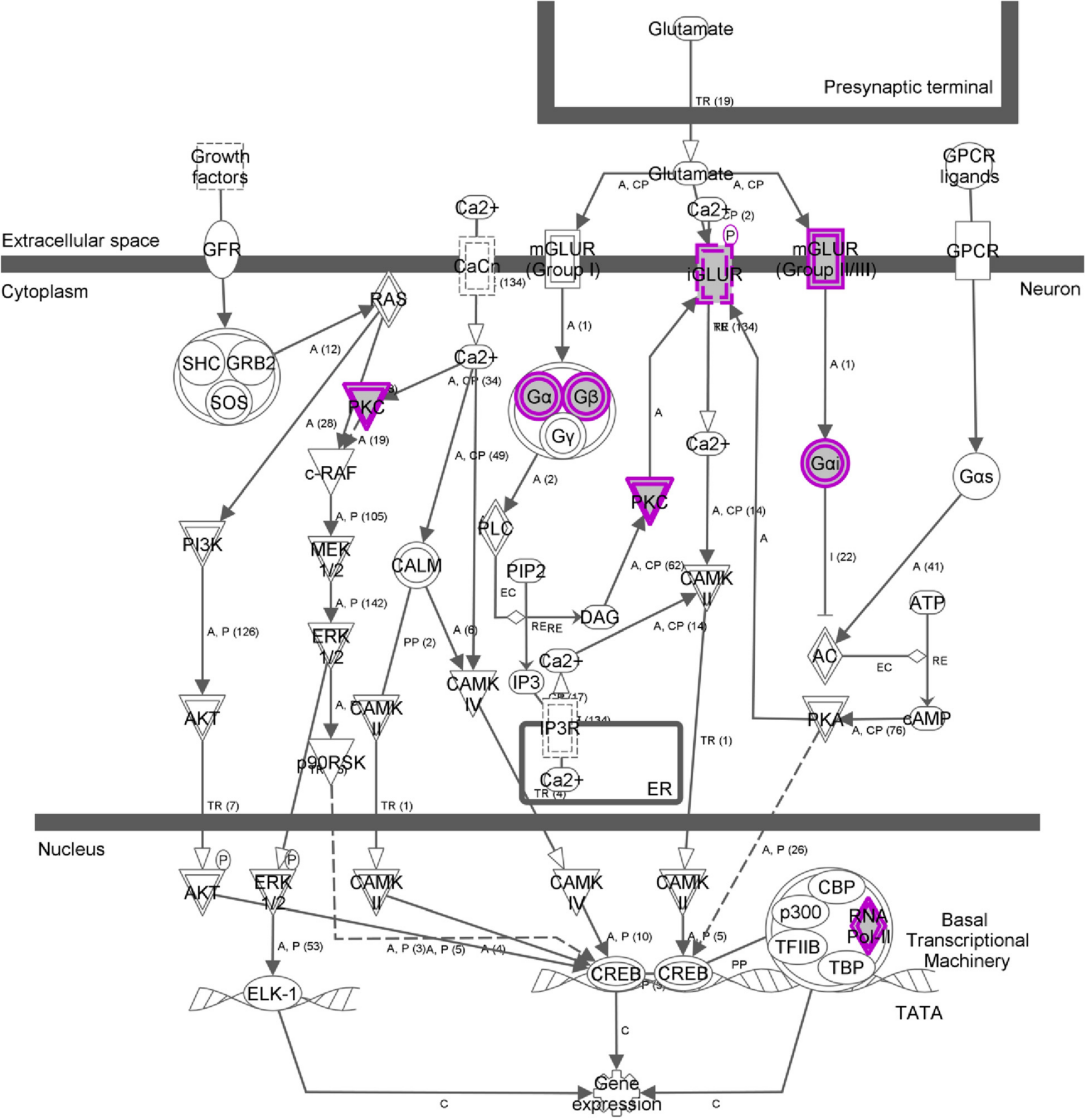
CREB signaling in neurons (genes hypomethylated in the schizophrenia patient of Twin Pair 2)

It is apparent from the results included in Table 2 that promoter hypomethylation of genes in the patient significantly affects canonical pathways involving glutamate receptor signaling and CREB signaling in neurons (*p* = 1.53E-02) (Fig. 4), as well as molecular and cellular functions, specifically cell signalling (*p* = 4.34E-06–4.51E-02), nucleic acid metabolism (*p* = 4.34E-06–4.51E-02), and small molecule biochemistry (*p* = 4.34E-06–4.51E-02).

#### Shared pathways between olanzapine treated rat brains and affected twins

**Fig. 5:**
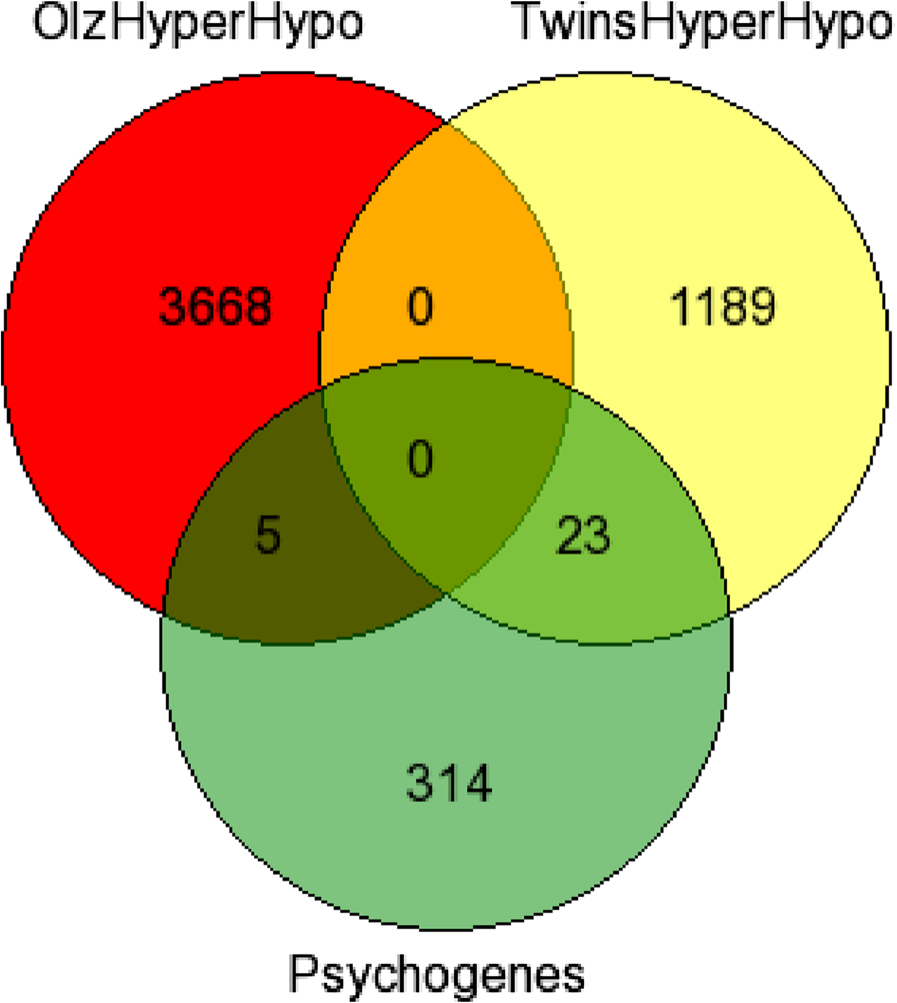
Venn diagram depicting the number of psychosis related genes (Psychogenes), hyper/hypomethylated genes in monozygotic twins discordant for schizophrenia (TwinsHyperHypo) and antipsychotic drug (olanzapine) treated rat brains (OlzHyperhypo). The 5 shared genes included: Cspg4, Gstm1, Htr7, Pax6 and Stat3). List of the 23 psychosis-candidate genes that are found to be hyper/hypomethylated in twins is presented in Additional file 2: Table S3

A therapeutic dose of olanzapine caused hyper- and hypo-methylation of genes involved in several psychosis related pathways and networks. Further details on the data are available on Melka *et al*. [15]. Intriguingly, some of the most significant pathways identified in monozygotic twins discordant for schizophrenia were also affected in rat brain regions. The common pathways and networks affected by olanzapine-induced hypermethylation and the twin patients (exposed to antipsychotic drugs) included the MAPK signaling pathway (*p* = 1.59E-03), a cell death, cellular growth and proliferation network (peak score = 24) (Table 1, Additional file 1: Tables S1 and S2), and a molecular transport and protein trafficking (peak score = 12) network (Table 2, Additional file 1: Table S2). Similarly, hypomethylation of genes in both sets of results (i.e. olanzapine treated rat brains and the twin study) affected calcium signaling (*p* = 5.92E-03) (Table 1, Additional file 1: Table S1). We have noted, despite the fact that there are some commonly affected pathways and networks between the two sets of results, that the individual genes involved in hyper/hypomethylation are not identical in rat brains and twin patients. However, both sets of results show methylation changes on psychosis related genes (Fig. 5).

## Discussion

The genome-wide methylation results on two pairs of monozygotic twins discordant for schizophrenia offer a number of insights. First, the twins do differ in methylation patterns. It follows similar conclusions in a number of reports [16, 17]. Interestingly, these differences appear to accumulate over time, and may account for the changing transcriptome [12] as well as the discordance of monozygotic twins for a variety of phenotypes and disorders, particularly psychosis.

Second, the genes affected by hyper/hypomethylation of promoters are not random. Several genes with promoter hyper/hypomethylation in schizophrenia twins were found to be psychosis related (Fig. 5, Additional file 2: Table S3). Predominantly, these genes appear to play a role in pathways and networks that are relevant in the context of the disease in question. Such conclusions follow a number of reports where discordance for psychosis has been suggested to involve differences in methylation [18]. This study has shown hyper- and hypo-methylation of genes affecting psychotic relevant pathways such as HIPPO signaling and *Ephrin* signaling, respectively, in twin pair 1. Similarly, hypermethylation of genes affecting Triacylglycerol Biosynthesis which had been associated with psychosis [19, 20]; and hypomethylation of genes affecting glutamate and CREB signaling pathways in twin pair 2, which are also associated with psychosis [21]. Interestingly, neural stem cell proliferation has been previously associated with altered methylation of CREB [22]. In both cases, the identified pathways are involved in disorders that may affect the development and course of psychosis. Further, it has been shown that epigenetic aberrations including methylation, may lead to disturbance of inhibitory activity and cortical associative functions [23]. Interestingly, hippocampal functions and cognitive performance could be influenced by epigenetic regulation of genes implicated in glutamate signaling [24]. The results support the relevance of developing personalized medicine not only with respect to genetic variations but also to epigenetic variations.

Third, the two twin pairs representing discordance for schizophrenia in two unrelated families show overlaps in pathways and interactions identified in the two patients. Of special interest to our findings are networks affected by hypermethylation of genes affecting Cell Death and Survival, Cellular Growth and Cellular Movement (Tables 1 and 2) in both schizophrenia affected patients. Similarly, genome-wide DNA methylation variations between adolescent monozygotic twins were associated with enrichment in pathways and networks involved in cellular growth and proliferation [25] and these significant differences in methylation of monozygotic twins have been associated with psychosis [26].

Finally, it is logical to ask the question regarding the cause of these differences. Is DNA methylation causing the disorder or is this due to other factors? To the best of our understanding this question has not been addressed satisfactorily, which is the focus of this section of the discussion. As mentioned, we have generated data on rats to further address this question. It involves the use of a rat model where a therapeutic dose of olanzapine is administered [13]. The results show that olanzapine affects methylation of a large number of genes [13]. They affect pathways and interactions that are compatible with psychosis. For example, we report epigenetic alterations of the dopaminergic system [27, 28] and serotonin transporter gene promoters [29], which have been evident in major psychiatric disorders, including schizophrenia [30]. In particular, hyper/hypomethylation in rat brain regions support the dopamine hypothesis of schizophrenia reflecting the effect of olanzapine [13]. This argument has been supported by two observations. First, common networks such as cellular growth and cellular movement were affected by hyper/hypomethylation in both patients. Second, psychotic-relevant pathways such as the Dopamine-*DARPP32* feedback in cAMP signaling were also affected by olanzapine-induced methylation [15]. Interestingly, those pathways have also been observed in the current twin study. We acknowledge that these results are based on only two pairs of MZ twins and there may be a need to confirm the results using additional methods such as bisulphite sequencing. However, the uniformity of the results being reported prompted us to ask the question, could methylation changes in the affected twin be caused by antipsychotic drugs that have been administered during the course of the management of their psychotic symptoms? We conclude by suggesting that this possibility cannot be ruled out. The implication is that studies on DNA methylation on psychiatric disorders where patients are often on medication should be interpreted carefully. More importantly, it may be premature for published reports to conclude that epigenetic changes are causes of psychosis in patients.

## Conclusion

First, DNA methylation differences between monozygotic twins discordant for schizophrenia revealed common networks affected in both unrelated twin pairs suggesting that those networks may underlie the cause of the disease. Although there exist common pathways, this study also showed that unique genes were affected in each pair of twins suggesting that the aetiology and/or the pathophysiology of the diseases may involve unique (patient-specific) genes entailing the need for personalized medicine in treating the disorder. Importantly, these patient-specific genes together with the observed effect of antipsychotics on genes in rat brains as compared to monozygotic twins corroborate the known heterogeneous nature of the disorder. Second, the results also showed patient-specific pathways, which support previous findings highlighting that the effects of antipsychotic drugs administered to patients may have caused at least some of the hyper/hypomethylation differences. These results reflect the fact that personalized medicine is not only necessary for patients of wider genetic variations but also for patients of epigenetic variations such as DNA methylation. The findings could be relevant for future therapeutic investigation of complex disorders in addition to schizophrenia.

## Availability of supporting data

Additional files, supporting the observations and discussion, are presented in Table S1, Table S2 and Table S3.

## Additional files

Additional file 1: Table S1. Most significant pathways and networks identified by Ingenuity pathway analysis using genes that showed hypermethylation (a) or hypomethylation (b) in hippocampus, following a therapeutic dose of olanzapine treatment in a rat model *in vivo*. **Table S2.** Most significant pathways and networks identified by Ingenuity pathway analysis using genes that showed hypermethylation (a) or hypomethylation (b) in cerebellum, following therapeutic dose of olanzapine treatment in a rat model *in vivo*. Additional file 2: Table S3. List of genes hypermethylated or hypomethylated in patient 1 and patient 2 relative to their respective healthy co-twins.
