# Supplementary material for: Insights into the origin of DNA methylation differences between monozygotic twins discordant for schizophrenia
Source: J Mol Psychiatry. 2015 Jun 26;3(1):7. doi: 10.1186/s40303-015-0013-5 (PMC4487197; doi:10.1186/s40303-015-0013-5)
Supplement: Additional file 1: Table S1. — Most significant pathways and networks identified by Ingenuity pathway analysis using genes that showed hypermethylation (a) or hypomethylation (b) in hippocampus, following a therapeutic dose of olanzapine treatment in a rat model in vivo. Table S2. Most significant pathways and networks identified by Ingenuity pathway analysis using genes that showed hypermethylation (a) or hypomethylation (b) in cerebellum, following therapeutic dose of olanzapine treatment in a rat model in vivo. [file 40303_2015_13_MOESM1_ESM.doc]

**Additional file 1: tables S1 and S2**

**Table S1. Most significant pathways and networks identified by Ingenuity pathway analysis using genes that showed hypermethylation (a) or hypomethylation (b) in hippocampus, following therapeutic dose of olanzapine treatment in a rat model *in vivo***

| **(a) Canonical Pathways** | **p-value** | **# Molecules^1^** | |
| --- | --- | --- | --- |
| Dopamine-DARPP32 Feedback in cAMP Signaling | 1.65E-03 | 20/157 (0.127) | |
| Role of JAK2 in Hormone-like Cytokine Signaling | 3.38E-03 | 7/34 (0.206) | |
| **Associated Network Functions** |  |  | |
| Metabolic Disease, Endocrine System  and developmental Disorders | | 35 | |
| Cell Cycle, Cellular Growth and Proliferation, Cell Death | | 24 | |
| **(b) Canonical pathways** | **p-value** | | **# Molecules** |
| Calcium Signaling | 5.92E-03 | | 12/178 (0.067) |
| D-myo-inositol (1,3,4,5,6)-Tetrakisphosphate Biosynthesis | 6.18E-03 | | 8/48 (0.167) |
| **Associated Network Functions** | | |  |
| Developmental Disorder, Cell Death and Survival, Cellular  Development | | | 12 |
| Molecular Transport, Nervous System Development  and Function | | | 10 |
| Carbohydrate Metabolism, Cell Morphology, Lipid Metabolism | | | 9 |

^1^For the top canonical pathways, ratio value (number of molecules in a given pathway that suffice the cut-off (p<=0.01) divided by the total number of molecules in the pathway).

**Table S2. Most significant pathways and networks identified by Ingenuity pathway analysis using genes that showed hypermethylation (a) or hypomethylation (b) in cerebellum, following therapeutic dose of olanzapine treatment in a rat model *in vivo***

| **(a) Canonical pathways** | **p-value** | **# Molecules^1^** |
| --- | --- | --- |
| Ephrin Receptor Signaling | 5.23E-04 | 24/176 (0.136) |
| MAPK Signaling | 1.59E-03 | 24/184 (0.13) |
| Protein Kinase A Signaling | 2.61E-03 | 41/372 (0.11) |
| **Associated Network Functions** | |  |
| Molecular Transport, Protein Synthesis, Protein Trafficking | | 12 |
| Behavior, Nervous System Development and Function | | 11 |
| **(b) Canonical pathways** | **p-value** | **# Molecules** |
| Ephrin B Signaling | 4.0E-03 | 7/72 (0.097) |
| G Beta Gamma Signaling | 4.1E-03 | 8/99 (0.081) |
| **Associated Network Functions** |  |  |
| Cell Death and Survival, Cellular Development | | 14 |
| Lipid Metabolism, Small Molecule Biochemistry | | 14 |
| Neurological Disease, Cellular Function and Maintenance, Molecular Transport | | 11 |

^1^For the top canonical pathways, ratio value (number of molecules in a given pathway that suffice the cut-off (p<=0.01) divided by the total number of molecules in the pathway).
